# Effect of novel antimicrobial blue light-emitting optical fiber on vancomycin-resistant *Enterococcus faecium* and carbapenemase-producing *Klebsiella pneumoniae*

**DOI:** 10.5194/jbji-10-561-2025

**Published:** 2025-12-09

**Authors:** Megan H. Goh, Barbara Körber-Irrgang, Lucy L. Hederick, Robert A. Rabiner, Hilmar Wisplinghoff, Antonia F. Chen, Nathalie Jazmati, Santiago A. Lozano-Calderon

**Affiliations:** 1 Department of Orthopaedic Surgery, Massachusetts General Hospital, Harvard Medical School, Boston, 02114, USA; 2 Wisplinghoff Laboratories, Cologne, Germany; 3 ABL Medical Inc., East Providence, 02914, USA; 4 Institute for Virology and Medical Microbiology, Witten/Herdecke University, Witten, Germany; 5 Department of Orthopaedic Surgery, University of Texas Southwestern Medical Center, Dallas, 75390, USA; 6 Institute for Medical Microbiology, Immunology and Hygiene, University Hospital of Cologne, Cologne, Germany

## Abstract

Periprosthetic joint infections (PJIs), particularly those caused by multidrug-resistant organisms (MDROs), remain a major therapeutic challenge. Antimicrobial blue light (ABL) offers a promising non-antibiotic approach, inducing bacterial killing through photoexcitation of endogenous chromophores and subsequent reactive oxygen species generation. However, conventional single-point illumination systems are limited by uneven light distribution and poor penetration, restricting their use to superficial infections. We evaluated a novel isotropic optical fiber designed to overcome these geometric and optical constraints. The fiber was tested against vancomycin-resistant *Enterococcus faecium* (VR-Ef) and carbapenemase-producing *Klebsiella pneumoniae* (CP-Kp) in time-to-kill assays under low-power (20.1 mW mm^−1^) and high-power (40.3 mW mm^−1^) conditions over 60 min. Bacterial counts (CFU per mL) were determined at 0, 10, 20, 30, and 60 min. A one-way analysis of variance (ANOVA) with Tukey's post hoc test assessed time-dependent reductions; a two-way ANOVA evaluated the combined effects of illumination power and exposure time. ABL exposure resulted in time- and intensity-dependent bacterial reduction in both strains. Significant CFU reductions occurred from 30 min onward under high-power ABL (HP-ABL) and after 60 min under low-power ABL (LP-ABL) for both VR-Ef and CP-Kp ( p<0.001 ). The two-way ANOVA revealed significant main and interaction effects of illumination power and exposure time (all p<0.001 ). Although bactericidal thresholds ( $\geq 3{\mathrm{log}}_{10}$ reduction) were not reached, bacterial killing increased markedly with higher power and longer exposure. This novel isotropic optical fiber enables uniform intraluminal ABL delivery, potentially extending blue-light therapy from superficial to deep surgical infections such as PJIs. Further optimization of illumination parameters and potential integration with photosensitizers may enhance its antimicrobial efficacy and clinical applicability.

## 1 Introduction

An increase in the number of total knee and hip arthroplasties has led to a corresponding rise in periprosthetic joint infections (PJIs) (Kurtz et al., 2007), which are associated with significant morbidity and mortality, prolonged hospital stays, and escalating healthcare costs (Bruyninckx et al., 2024). The current gold standard for treating PJIs, particularly in chronic cases, is two-stage revision arthroplasty (Charette and Melnic, 2018; Lazic et al., 2021). The first stage involves thorough debridement, removal of all prosthetic components, and implantation of an antibiotic-loaded cement spacer. This is followed by a period of systemic antibiotic therapy before the second stage, in which the spacer is removed and new prosthetic components are implanted (Parvizi et al., 2012). However, reinfection remains a persistent challenge, particularly in cases involving multidrug-resistant organisms (MDROs), with recurrence rates ranging from 24 % to 82 % (Whiteside et al., 2011). The difficulty in eradicating these infections largely stems from microbial biofilm formation on implant surfaces and limited intra-articular penetration of systemic antibiotics (Antony et al., 2015). Biofilms create a protective niche through a matrix of extracellular polymeric substances, shielding bacteria from both environmental stressors and antibiotics (Sharma et al., 2019).

Alarmingly, the widespread use and misuse of antibiotics have driven the emergence of MDROs, which have outpaced the development of new antimicrobial agents (Muteeb et al., 2023). Though rare, PJIs caused by highly resistant strains of *Klebsiella pneumoniae* and *Enterococcus faecium* can lead to devastating clinical outcomes, including numerous revision surgeries, extensive salvage procedures such as amputation, and death (Omichi et al., 2023; Piuzzi et al., 2024; Ries, 2001; de Sanctis et al., 2014; Sidhu and Antony, 2017). The incidence of multidrug-resistant *K. pneumoniae* infections has been steadily increasing worldwide, with limited treatment options contributing to mortality rates as high as 40 %–50 % in affected patient populations (Bassetti et al., 2018). Similarly, the extensive use of vancomycin has facilitated the rise of vancomycin-resistant *Enterococcus* (VRE), which is now the second most common nosocomial infection in the United States (Ries, 2001; Si et al., 2017). With few effective antibiotics available, VRE infections, largely comprising *Enterococcus faecium*, are associated with mortality rates ranging from 32 % to 66.7 % (Hemapanpairoa et al., 2021). Clearly, the growing threat of antimicrobial resistance (AMR) underscores the urgent need for improved antibiotic stewardship and the development of alternative, non-antibiotic adjuvant therapies.

Antimicrobial blue light (ABL) has emerged as a promising non-antibiotic approach with bactericidal effects. Exposure of bacterial cells to visible light wavelengths between 400 and 470 nm induces the production of reactive oxygen species (ROS) through the photoexcitation of endogenous porphyrins and/or flavins (Leanse et al., 2022). This leads to microbial death via damage to essential structures such as DNA, cell membranes, and proteins (Leanse et al., 2022).

The extent of ROS production, however, depends on the presence and abundance of such chromophores, which vary among bacterial species. While porphyrin-mediated ROS formation has been demonstrated in several Gram-positive and Gram-negative bacteria, some species – such as *Enterococcus* spp. – do not appear to accumulate detectable porphyrins (Frankenberg et al., 2002), suggesting that alternative endogenous photosensitizers (e.g., flavins or NADH) may contribute to blue-light susceptibility (El-Gendy et al., 2024). In contrast, *Klebsiella pneumoniae* can exhibit detectable porphyrin-associated fluorescence under violet–blue excitation, but endogenous photosensitizer levels appear to be strain- and condition-dependent, and the precise chromophores governing ROS generation remain incompletely characterized (Sun et al., 2025).

Importantly, mammalian host cells also contain porphyrin biosynthesis pathways but at markedly lower intracellular concentrations than many bacterial species, which contributes to the selective antimicrobial window of ABL. In vitro studies directly comparing ESKAPE pathogens (*Enterococcus faecium*, *Staphylococcus aureus*, *Klebsiella pneumoniae*, *Acinetobacter baumannii*, *Pseudomonas aeruginosa*, and *Enterobacter* species) and human cells have shown that a significant reduction in viable bacteria could be achieved by ABL at subtoxic irradiation doses, supporting a potential use of visible light as an antimicrobial agent in clinical settings (Bauer et al., 2021).

While the therapeutic effects of ABL have been explored in several preclinical studies using single-point illumination systems, its applications have been limited to superficial infections, such as those affecting the skin (Dai et al., 2012, 2013; Negri et al., 2024; Wang et al., 2016) and mouth (Chui et al., 2012; Song et al., 2013; Yoshida et al., 2017). This limitation arises from several fundamental optical and practical constraints. ABL delivered through single-point illumination (Mohamad et al., 2021; Tsutsumi-Arai et al., 2022) or its modified variants (Bumah et al., 2020b; Felix Gomez et al., 2019) is limited by the attenuation of light intensity over distance, making it impractical for treating luminal surfaces (i.e. the intra-articular surfaces of joints, body cavities, or conduits). According to the inverse square law, light intensity decreases at a rate proportional to the square of the distance between the emitting source and treatment area, resulting in significant reductions in effective illumination over large areas (Brownson, 2014). Therefore, illumination of a cavity with a single point would result in an uneven ABL dosing to the desired treatment zone, with areas further from the emitting source receiving substantially less ABL exposure. Additionally, there would be navigational and directional challenges to ensuring that all areas of the infection zone were sufficiently treated with a single-point ABL illumination device. As a result, the treatment of deep infections such as PJIs poses a fundamentally different and more challenging engineering problem, which single-point illumination systems are ill suited to tackle. To address the limitations of single-point illumination, a novel, isotropic optical fiber was designed to radially emit ABL with equal power and light intensity along the entirety of the active fiber length. Our prior work (Goh et al., 2025a, b; Körber-Irrgang et al., 2023a, b) with this new fiber demonstrated promising bactericidal effects on antibiotic-susceptible and multidrug-resistant *Pseudomonas aeruginosa*, methicillin-susceptible and methicillin-resistant *Staphylococcus aureus*, and antibiotic-susceptible *Escherichia coli*, as well as clinically relevant reductions in extended-spectrum beta-lactamases *Escherichia coli*.

In this study, we evaluated the potential of a novel optical fiber as a non-antibiotic adjuvant therapy for PJIs caused by carbapenemase-producing *Klebsiella pneumoniae* (CP-Kp) and vancomycin-resistant *Enterococcus faecium* (VR-Ef). Specifically, we assessed the in vitro antimicrobial activity of blue light emitted by the fiber against these MDROs.

## 2 Materials and methods

### 2.1 Optical fiber design and blue-light source

**Figure 1:**
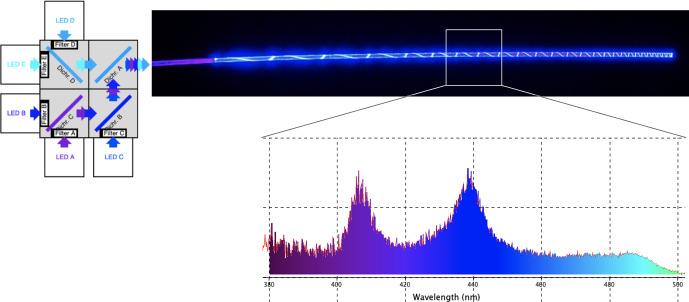
Schematic representation of the blue-light-source LED design and multiwavelength output from the optical fiber.

A detailed description of the optical fiber design and blue light source was presented in prior work (Goh et al., 2025a, b). Briefly, a polymer optical fiber (POF) was developed using a polymethyl methacrylate (PMMA) core coated with a fluorinated polymer cladding (20 µ m thick) to enable efficient visible light transmission with minimal optical loss. The POF is isotropic, meaning that it has a uniform refractive index in all directions. This property reduces uneven scattering and enables consistent, circumferential light distribution along the fiber, which is essential for uniform tissue illumination. By leveraging total internal reflection, the PMMA core functioned as a transmissive fiber in accordance with Snell's law. To achieve uniform ABL distribution along the fiber's entire active length, sections of the cladding were incrementally removed in a helical pattern, allowing both linear and 360° radial light emission without significant power loss over distance. A custom-designed light motor facilitated the delivery of multiple ABL wavelengths (405–475 nm) through the fiber, utilizing dichromic mirrors to reflect and combine noncoherent light from five high-powered LEDs (405, 415, 435, 450, and 475 nm) into a single output (Fig. 1). This system enabled precise modulation of optical power, allowing controlled light exposure.

### 2.2 Bacterial suspension preparation

All experiments were carried out at Wisplinghoff Laboratories, Cologne, Germany, an accredited medical laboratory. The bacterial strains of *K. pneumoniae* AR1045 (carbapenemase-producing, KPC-2) (CP-Kp) and *E. faecium* BLS-1 (vancomycin-resistant, VanA) (VR-Ef) were obtained from commercial bacterial suppliers (Leibniz Institute DSMZ; CDC and FDA Antimicrobial Resistance Isolate Bank).

One or more colonies from an overnight culture of the respective bacterial strain were mixed with 2 mL of NaCl 0.85 % (API^®^ medium, bioMérieux Inc.). Suspension turbidity was measured using a densitometer to determine the bacterial concentration according to McFarland standard (Lahuerta Zamora and Pérez-Gracia, 2012). The target turbidity was 0.5 McF, corresponding to approximately 1.5×108 CFU per mL (Balouiri et al., 2016; Leber, 2016). Suspensions were adjusted as needed by adding either more bacterial colonies from the respective overnight culture or more NaCl.

The adjusted bacterial suspension of a respective strain was further diluted in NaCl to obtain an inoculum of approximately 1.0×105 CFU per mL. Immediately after inoculum preparation, 10 mL each of the bacterial inoculum was then transferred into three test tubes for immediate subsequent testing.

### 2.3 Time-to-kill assays

**Figure 2:**
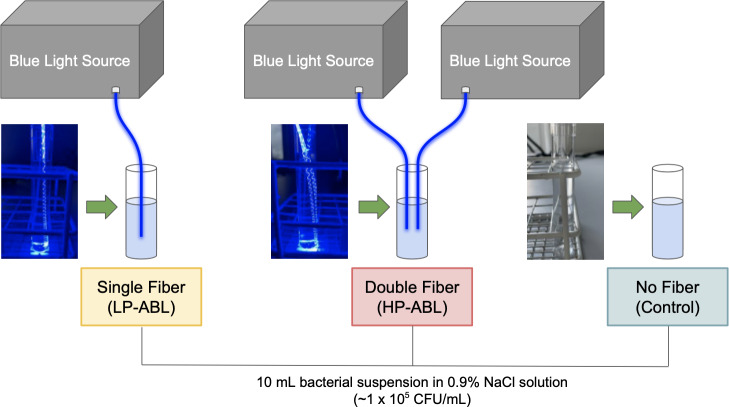
Experimental setup for time-to-kill assays. LP-ABL and HP-ABL were delivered with the placement of one optical fiber and two optical fibers, respectively, within the test tube containing the bacterial suspension. No optical fiber was placed within the bacterial suspension of the control tube. All test tubes contained a 10 mL bacterial suspension of $∼1\times 10^{5}$ CFU per mL.

Three study groups were evaluated based on the level of ABL exposure: (1) low-power ABL (LP-ABL; one optical fiber, 20.1 mW mm^−1^), (2) high-power ABL (HP-ABL; two optical fibers, 40.3 mW mm^−1^), and (3) a control group with no ABL exposure. ABL was delivered using the novel optical fiber (1.5 mm diameter, 10 cm active light-emitting length). In the experimental groups, optical fiber(s) were inserted into test tubes and secured with flexible arms (Fig. 2). The optical fibers were submerged such that the entire height of the bacterial suspension would be exposed to ABL. All time-to-kill assays were performed at ambient room temperature.

Each fiber simultaneously emitted five continuous ABL wavelengths (405, 415, 435, 450, and 475 nm) for 60 min. Samples were collected at five time points (0, 10, 20, 30, and 60 min) from both experimental and control groups and were directly diluted 1:10 and 1:100 . 50 µ L of the respective dilution and 50 µ L of the undiluted sample were streaked onto Mueller–Hinton agar plates by a spiral plater (EddyJet, IUL Instruments). Plating was performed in triplicate. The plates were incubated at 36±1 °C for up to 36 h. The CFU per mL for each time point was determined as the mean of triplicate plate counts. Time-to-kill assays were conducted eight times per bacterial strain (twice each on 4 separate days). A bactericidal effect was defined as a ≥99.9 % ( $\geq 3{\mathrm{log}}_{10}$ ) reduction in CFU per mL (Zadrazilova et al., 2015).

### 2.4 Heat dissipation measurements

Measurements of the potential heat generated from the novel optical fiber(s) in the LP-ABL (one optical fiber, 20.1 mW mm^−1^) and HP-ABL (two optical fibers, 40.3 mW mm^−1^) and from an additional extra-high-power ABL (XHP-ABL; three optical fibers, 60.3 mW mm^−1^) group were conducted to ensure safety of use. These experiments and the respective results were described in our prior work (Goh et al., 2025a, b).

### 2.5 Statistical analysis

To evaluate the effect of exposure time on bacterial reduction within each illumination intensity (LP-ABL and HP-ABL), a one-way analysis of variance (ANOVA) was conducted using baseline-normalized ${\mathrm{log}}_{10}$ (CFU per mL reduction) values as the dependent variable. Because the baseline ( t=0 min) was already incorporated into the reduction values, post hoc pairwise comparisons between time points were performed using Tukey's honestly significant difference (HSD) test. To assess the combined effects of exposure time and illumination intensity, a two-way ANOVA was applied with exposure time (10, 20, 30, 60 min) and illumination intensity (LP-ABL vs. HP-ABL) as fixed factors. Statistical significance was set at p<0.05 for all comparisons. All statistical analyses were performed using R.

**Figure 3:**
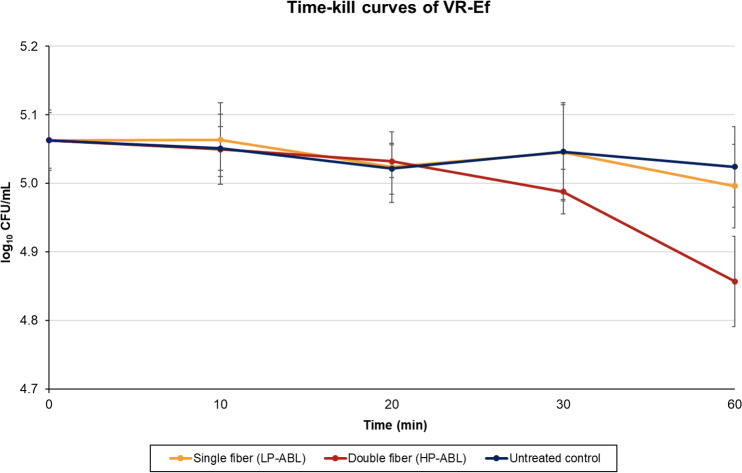
Time-to-kill curves for vancomycin-resistant *E. faecium*. Mean ${\mathrm{log}}_{10}$ CFU per mL values of eight LP-ABL and eight HP-ABL tests are displayed. The standard deviation is indicated as an error bar in the test series at each point in time.

## 3 Results

For VR-Ef, LP-ABL resulted in a ${\mathrm{log}}_{10}$ CFU per mL ± SD difference (percent reduction) of 0.00±0.03 ( +0.48 %), -0.04±0.05 (8.16 %), -0.02±0.06 (3.13 %), and -0.07±0.05 (13.80 %) at 10, 20, 30, and 60 min, respectively (Fig. 3). For HP-ABL, there was a ${\mathrm{log}}_{10}$ CFU per mL ± SD difference (percent reduction) of -0.01±0.03 (2.79 %), -0.03±0.05 (6.27 %), -0.07±0.04 (15.49 %), and -0.21±0.08 (36.79 %) at 10, 20, 30, and 60 min, respectively (Fig. 3). For VR-Ef, one-way ANOVA revealed a significant time effect for both LP-ABL ( p=0.049 ) and HP-ABL ( p<0.0001 ). Post hoc Tukey tests revealed that, under LP-ABL, bacterial counts were significantly reduced only after 60 min ( p=0.042 ), whereas under HP-ABL, significant reductions were observed from 30 min onward ( p<0.001 ). Two-way ANOVA confirmed significant main effects of time ( p<0.0001 ) and illumination intensity ( p=0.0004 ), as well as a significant interaction ( p=0.001 ), indicating that bacterial reduction progressed more rapidly and extensively under high-power illumination.

**Figure 4:**
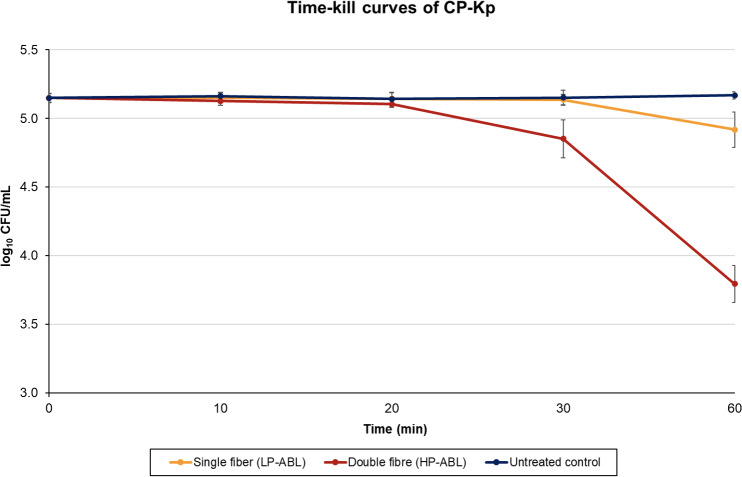
Time-to-kill curves for carbapenemase-producing *K. pneumoniae*. Mean ${\mathrm{log}}_{10}$ CFU per mL values of eight LP-ABL and eight HP-ABL tests are displayed. The standard deviation is indicated as an error bar in the test series at each point in time.

For CP-Kp, LP-ABL resulted in a ${\mathrm{log}}_{10}$ CFU per mL ± SD difference (percent reduction) of 0.00±0.04 ( +0.81 %), -0.01±0.05 (0.76 %), -0.01±0.05 (2.60 %), and -0.23±0.13 (38.94 %) at 10, 20, 30, and 60 min, respectively (Fig. 4). For HP-ABL, there was a ${\mathrm{log}}_{10}$ CFU per mL ± SD difference (percent reduction) of -0.02±0.04 (4.64 %), -0.04±0.04 (9.48 %), -0.30±0.13 (47.40 %), and -1.35±0.14 (95.36 %) at 10, 20, 30, and 60 min, respectively (Fig. 4). For CP-Kp, one-way ANOVA revealed a significant effect of exposure time for both LP-ABL ( p<0.0001 ) and HP-ABL ( p<0.0001 ). Post hoc Tukey tests showed that, under LP-ABL, significant reductions occurred only after 60 min ( p<0.001 ), whereas under HP-ABL, reductions became significant after 30 min and further intensified at 60 min (all p<0.001 ). Two-way ANOVA demonstrated significant main effects of exposure time ( p<0.0001 ) and illumination intensity ( p<0.0001 ), as well as a significant interaction between time and intensity ( p<0.0001 ), indicating a markedly stronger and faster antimicrobial effect under high-power illumination.

## 4 Discussion

In this study, we investigated the bactericidal potential of ABL, emitted by a novel isotropic optical fiber against CP-Kp and VR-Ef under in vitro conditions. ABL is hypothesized to facilitate microbial death through the photoexcitation of endogenous chromophores, triggering the generation of cytotoxic ROS and thus inducing multitargeted damage to critical microbial structures such as lipids, DNA, and cell membranes (Leanse et al., 2022). Importantly, ABL poses minimal risk of inducing or contributing to the rise in microbial resistance (Zhang et al., 2014).

The emergence of VRE has been strongly associated with the widespread clinical use of vancomycin (Rice, 2001). Resistance is conferred through the *van* gene operon, which reduces the binding affinity of vancomycin for target sites within the bacterial cell wall. Treatment options for VRE are limited due to the multifaceted resistance mechanisms – both intrinsic and acquired – that these bacteria possess, including structural and metabolic adaptations, horizontal gene transfer and genetic mutations, and the formation of biofilms that hinder antibiotic penetration and efficacy (Hota et al., 2025). While ABL has demonstrated promise in treating other MDROs (Bumah et al., 2020a; Dos Anjos et al., 2024; Fila et al., 2017; Zhang et al., 2014), there is limited research exploring its ability to fight VRE. El-Gendy et al. (2024) investigated antimicrobial photoactivation (aPI) in vancomycin-resistant *E. faecalis* using femtosecond single-point illumination lasers to deliver single blue-light wavelengths (420–465 nm) at a fixed fluence of 1000 J cm^−2^ (El-Gendy et al., 2024). Fluence, which quantifies the amount of energy delivered per unit area, contextualizes spatial energy concentration (Farkas and Geretovszky, 2006). The wavelengths of 430 and 435 nm achieved significant bacterial reductions of 98.6 % and 98.3 %, respectively (El-Gendy et al., 2024). Similarly, Hoenes et al. applied two distinct ABL wavelengths (405 nm at 825 J cm^−2^ and 450 nm at 1250 J cm^−2^) via single-point illumination to VR-Ef, achieving >99.9 % bacterial reduction (Hoenes et al., 2020). In contrast, our study achieved only a 36.79 % CFU reduction in VR-Ef following 60 min of HP-ABL exposure (using wavelengths of 405, 415, 435, 450, and 475 nm simultaneously). This discrepancy may be due to differences in bacterial strains, with our particular strain of *E. faecium* exhibiting greater ABL resistance potentially stemming from reduced porphyrin production, a critical step in the antimicrobial mechanism of action for ABL (Jones et al., 2020). Furthermore, while previous studies applied single-point illumination and single-wavelength treatments, our fiber delivered ABL circumferentially across multiple simultaneous wavelengths. Specifically, at 60 min of HP-ABL exposure, the total estimated fluence of the optical fiber was 967 J cm^−2^; however, this energy was distributed across five wavelengths rather than concentrated into a single-point, single high-intensity wavelength as in El-Gendy et al. (2024) and Hoenes et al. (2020). Although our study included the similar effective range of wavelengths reported by El-Gendy et al. (2024) and Hoenes et al. (2020), the energy dose per wavelength may have been insufficient for optimal inactivation of VR-Ef. Furthermore, neither study clearly specified the duration of ABL exposure, making direct comparisons to our experimental design challenging. While our experimental groups did not exhibit a bactericidal effect, further optimization of ABL treatment parameters – including fluence distribution, intensity, and exposure duration – may enhance its therapeutic efficacy against VRE.

The global rise of CP-Kp has become a significant public health concern, with antibiotic resistance conferred through bacterial plasmid acquisition of carbapenemase genes or the production of extended-spectrum beta-lactamases (ESBLs) combined with porin expression alterations (Budia-Silva et al., 2024; Tesfa et al., 2022). Given the limited treatment options for multidrug-resistant *K. pneumoniae*, ABL has been explored as a potential alternative therapy (Dos Anjos et al., 2020a, b). Dos Anjos et al. (2020b) demonstrated bacterial CFU reductions exceeding 3 ${\mathrm{log}}_{10}$ for five strains of hypervirulent and hyperviscous *K. pneumoniae* following 180 min of single-point illumination, single-wavelength ABL (410 nm at 1080 J cm^−2^) (Dos Anjos et al., 2020b). In a subsequent study, Dos. Anjos et al. (2020a) achieved >3 ${\mathrm{log}}_{10}$ reductions in ESBL-producing *K. pneumoniae* and CP-Kp strains with single-point illumination, single-wavelength ABL (410 nm at fluences of 836.87 and 892.28 J cm^−2^, respectively) after 139 and 148 min of treatment, respectively (Dos Anjos et al., 2020a). While our study did not reach the bactericidal threshold for CP-Kp with either LP-ABL or HP-ABL, a 60 min HP-ABL treatment achieved a 95.36 % reduction in bacterial CFUs, which may still be considered clinically relevant. Further optimization of ABL treatment parameters may enhance its effectiveness, supporting its potential as an adjunctive therapy to reduce bacterial loads in combination with existing treatment strategies.

This study evaluating a novel ABL-emitting optical fiber did not achieve a bactericidal reduction in the tested strains of VRE and CP-Kp. While the antibacterial reductions observed in this study appear lower than other published reports, direct comparisons are limited as prior work has relied on single-point illumination systems, which are not directly translatable to the circumferential fiber design evaluated here. The more complex illumination geometry may partially explain the differences observed, and optimization of exposure time and intensity will be essential to fully realize the antibacterial potential of this system. Extended exposure durations represent a key area for future work. However, the modest reduction in CFU counts – particularly in CP-Kp – suggests preliminary therapeutic promise. These findings highlight two important considerations: (1) the need to further optimize ABL delivery parameters and (2) the possibility that certain bacterial strains may exhibit greater resistance to ABL than others (Jones et al., 2020). As previously mentioned, the antimicrobial mechanism of ABL relies on the presence of endogenous bacterial chromophores called porphyrins, which facilitate the generation of ROS that mediate cell death (Leanse et al., 2022). However, some bacteria, such as certain strains of Enterococcus species, do not produce porphyrins (Jones et al., 2020). Without the production of porphyrins, these strains of bacterium are inherently more resistant to ABL and would require the use of exogenous chromophores, such as photosensitizers, to enhance antimicrobial efficacy (Bravo et al., 2024; Woźniak et al., 2021). Antimicrobial photodynamic therapy (aPDT), which couples light exposure with a photosensitizing agent, induces the generation of ROS and has shown synergistic effects against multidrug-resistant pathogens (Liu et al., 2015). Leveraging photosensitizers alongside ABL could therefore amplify its therapeutic effects. Future studies are needed to explore the combined approach of photosensitizers with this ABL-emitting optical fiber. Although this study was preclinical and focused on evaluating antibacterial activity against multidrug-resistant organisms, future work will be necessary to define how optical fiber treatment could be incorporated into surgical workflows. One potential application may be in staged revision arthroplasty, where fiber-based illumination could be deployed during the interim period between implant removal and reimplantation. Further studies will be required to assess feasibility, safety, and time management considerations in the operative setting.

This study had several limitations. First, as a preliminary in vitro proof-of-concept study, it does not fully capture the complexities of in vivo conditions. The in vitro design was intended to be clinically relevant in part by immersing the optical fiber directly into the bacterial suspension, which enables circumferential illumination that better approximates the potential treatment of an infected cavity, compared to traditional single-point external illumination models. Future studies will focus on developing physiologic models that better mimic endogenous in vivo environments. Second, we tested only a single strain of *K. pneumoniae* and *E. faecium*, limiting the generalizability of our findings to other MDROs outside of those we have already explored in this and prior research (Goh et al., 2025a, b). Future studies will aim to incorporate a broader range of MDRO strains to better assess the efficacy of this novel optical fiber across multiple bacterial species. Third, this study only evaluated two ABL intensities (LP-ABL and HP-ABL) for a maximal treatment duration of 60 min. Based on the test procedure in our prior published work (Goh et al., 2025a, b; Körber-Irrgang et al., 2023a, b), the duration of light exposure was not extended to determine whether a ≥3 log⁡ reduction could be achieved. Further research is needed to optimize both exposure time and intensity for maximal bactericidal efficacy.

## 5 Conclusion

ABL represents a promising therapeutic approach for treating PJIs caused by MDROs. While the current gold standard, two-stage revision arthroplasty, can effectively eradicate some prosthetic-related infections, the rising incidence of PJIs underscores the need for adjunctive therapies that mitigate the risk of further antibiotic resistance. With further research to optimize ABL delivery parameters in in vivo models across diverse MDRO strains, our novel optical fiber may have the potential to deliver ABL as an adjuvant treatment of luminal infections such as PJIs.

## Data Availability

All pertinent data have been made available in the paper.
